# Mediastinal Cartilaginous Hamartoma

**DOI:** 10.7759/cureus.7411

**Published:** 2020-03-25

**Authors:** Dejan C Vuckovic, Milos P Koledin, Nada M Vuckovic, Bojan M Koledin

**Affiliations:** 1 Department of Pathology, Faculty of Medicine, University of Novi Sad, Novi Sad, SRB; 2 Department of Pathology, Institute for Pulmonary Diseases of Vojvodina, Sremska Kamenica, SRB; 3 Clinic for Thoracic Surgery, Institute for Pulmonary Diseases of Vojvodina, Sremska Kamenica, SRB; 4 Pathology and Histology Centre, Clinical Centre of Vojvodina, Novi Sad, SRB

**Keywords:** lung, hamartoma, mediastinum

## Abstract

Pulmonary hamartomas are usually solitary, nodular benign lesions in the parenchyma of the lung. They are rarely situated in endobronchial areas, and very few cases are reported with the mediastinum.

A 56-year-old female patient got a CT-scan conducted due to coughing and breathlessness and was diagnosed with a nodular lesion in the medial mediastinum. The lesion was operated: it measured up to 4 cm in the largest diameter, had a smooth surface, was of rather soft but elastic consistency, and was extirpated. At pathology, on cut section, it was yellowish and lobular, and with a mixture of cartilaginous, fibrous and adipose tissues with some smooth muscle cell fibers and myxoid areas.

The diagnosis of pulmonary hamartoma was made with atypical medial mediastinal localization. This rare presentation could pose some differential diagnostic problems in the clinical diagnosis of more frequent primary and metastatic malignant diseases.

## Introduction

A hamartoma is a benign growth or focal malformation of an abnormal mixture of cells and tissues that is normally found in the area of the body where growth occurs, but in a disorganized manner. Although they are considered developmental malformations, many hamartomas have clonal chromosomal aberrations that are now considered to be neoplastic. In favor of the malformation theory, stands the fact that they have the same growth rate as the surrounding tissue. Hamartomas could occur in many parts of the body, and usually, as an asymptomatic mass, they are diagnosed as incidentalomas (found on an imaging study for some other reason).

They are closely related to choristoma, which is defined as a mass of tissue growing at the abnormal site. For both lesions, the underlying reasons for their occurrence are still not completely understood.

The most common place for hamartoma is the lung, where it comprises about 5-8% of all solitary nodules and up to 75% of all benign tumors. By definition, histological components of hamartoma are at least two mesenchymal tissues, mainly cartilage, mixed with adipose cells or some other cell types (fibrous or smooth muscle), and bronchial epithelium [1]. Hamartomas are rarely almost completely composed of adipose tissue. The majority of hamartomas are in the peripheral part of the lungs, approximately 10% are in deep lung tissue, and less than 10% are endobronchial [1].

Lung hamartomas are more frequent in men than in women, with the peak incidence of diagnosis in the 50s and 60s, and although usually asymptomatic, they may present some difficulties in smokers [1].

## Case presentation

**Figure 1:**
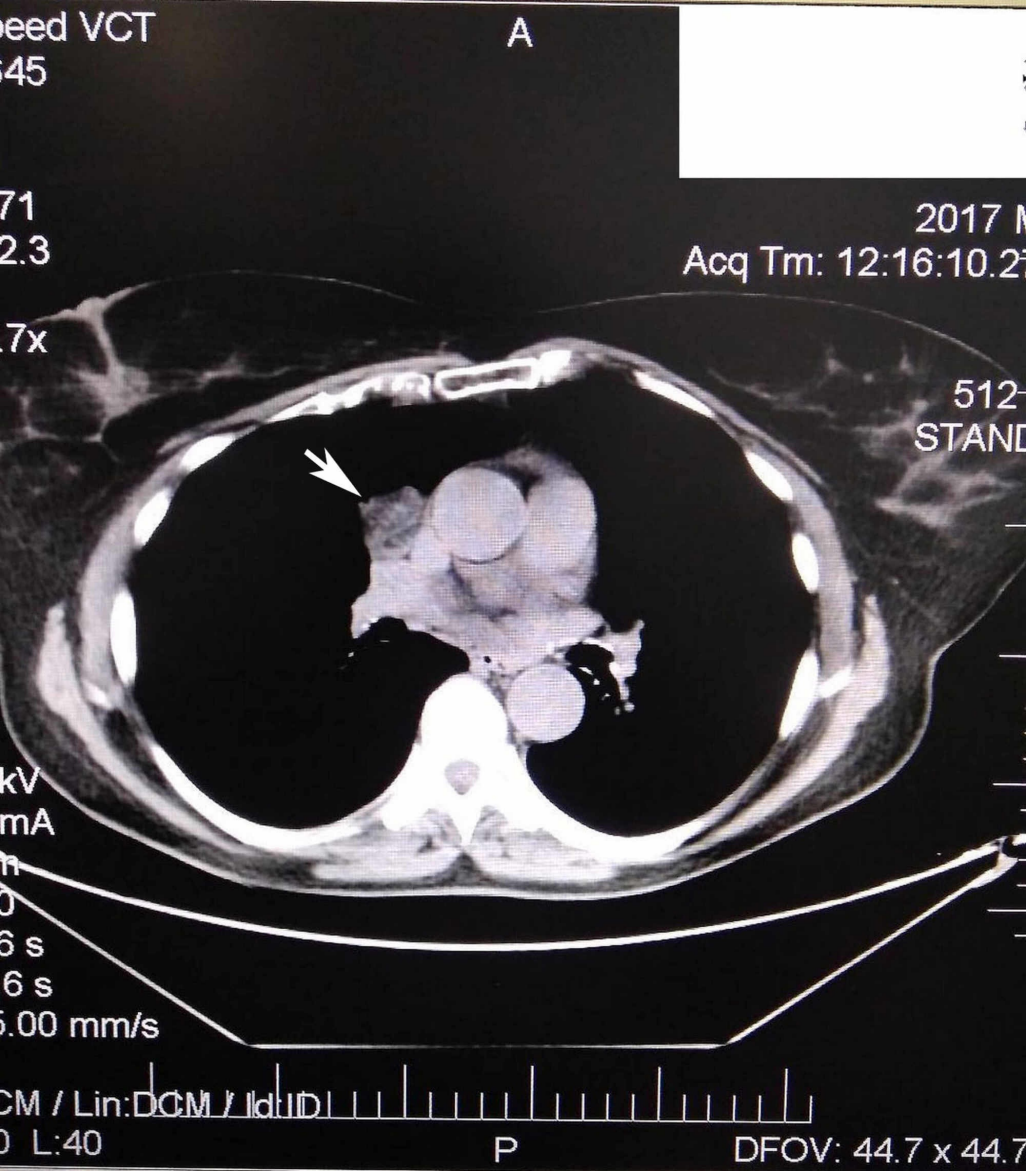
CT-scan showing the oval mass in the right side medial mediastinum

**Figure 2:**
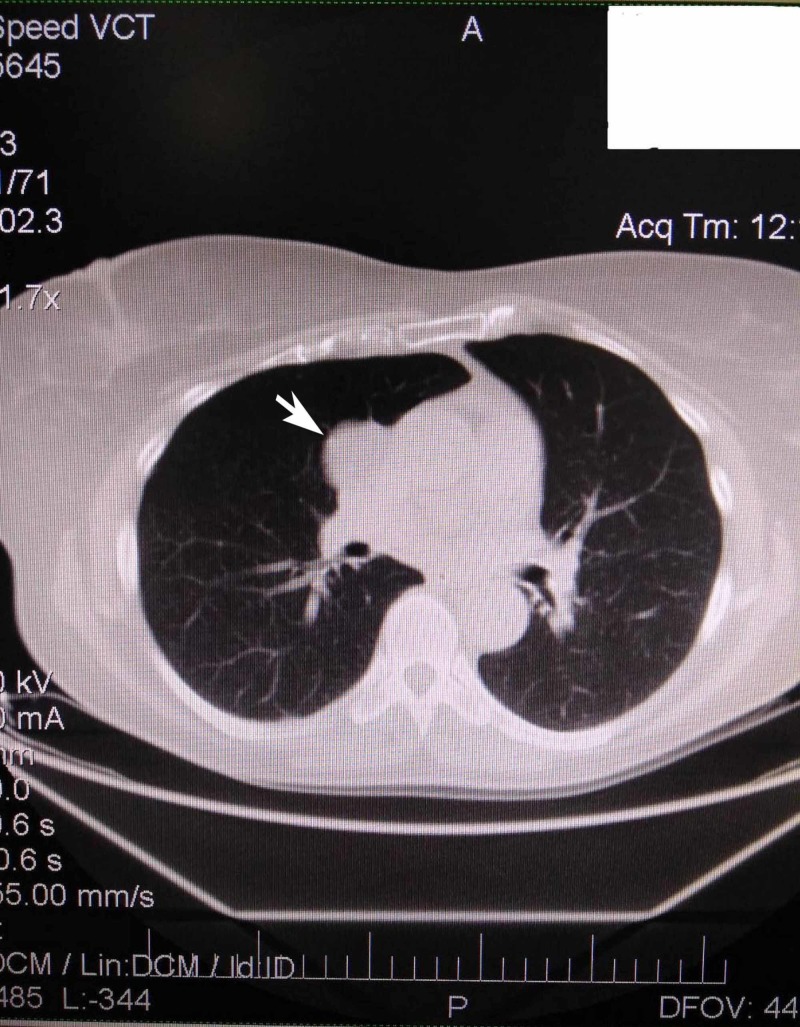
Enhanced CT-scan with a solitary lesion in the right side medial mediastinum

A 56-years-old female patient who was previously a heavy smoker for 15 years ceased for almost the last 20 years due to proven pollen allergy. Following the exacerbation of cough and breathlessness, a CT-scan was conducted, and a single mass in the medial mediastinum was diagnosed. The mass was situated in the right side of the thorax, close to the mediastinal pleura at the level of superior vena cava; it was oval and well circumscribed. It was hypodense, had an inhomogeneous appearance, and with measured Hounsfield values from +10 up to -10. The greatest dimensions measured were 38 x 30 mm; it was extrapulmonary and in contact with pericardium in its caudal part (Figures 1, 2). On both sides, the pleuropericardial adhesions were noticed. On latero-basilar left lung area, the ground glass appearance with micronodular hypodense changes was consistent with the inflammatory changes. Mediastinal and other lymph nodes and other organs were within normal limits. All laboratory values were within normal limits. Under general anesthesia, a video-assisted thoracoscopic surgery (VATS) was conducted wherein the mass was seen, and frozen sections were obtained for biopsy, which eventually confirmed the diagnosis of pulmonary hamartoma. After that, the anterolateral thoracotomy through the fifth right intercostal space was done with the complete excision of the mediastinal mass. In the pleural cavity, the drain of 28F was inserted. The operation and the postoperative period were uneventful, and the patient was released after four days.

**Figure 3:**
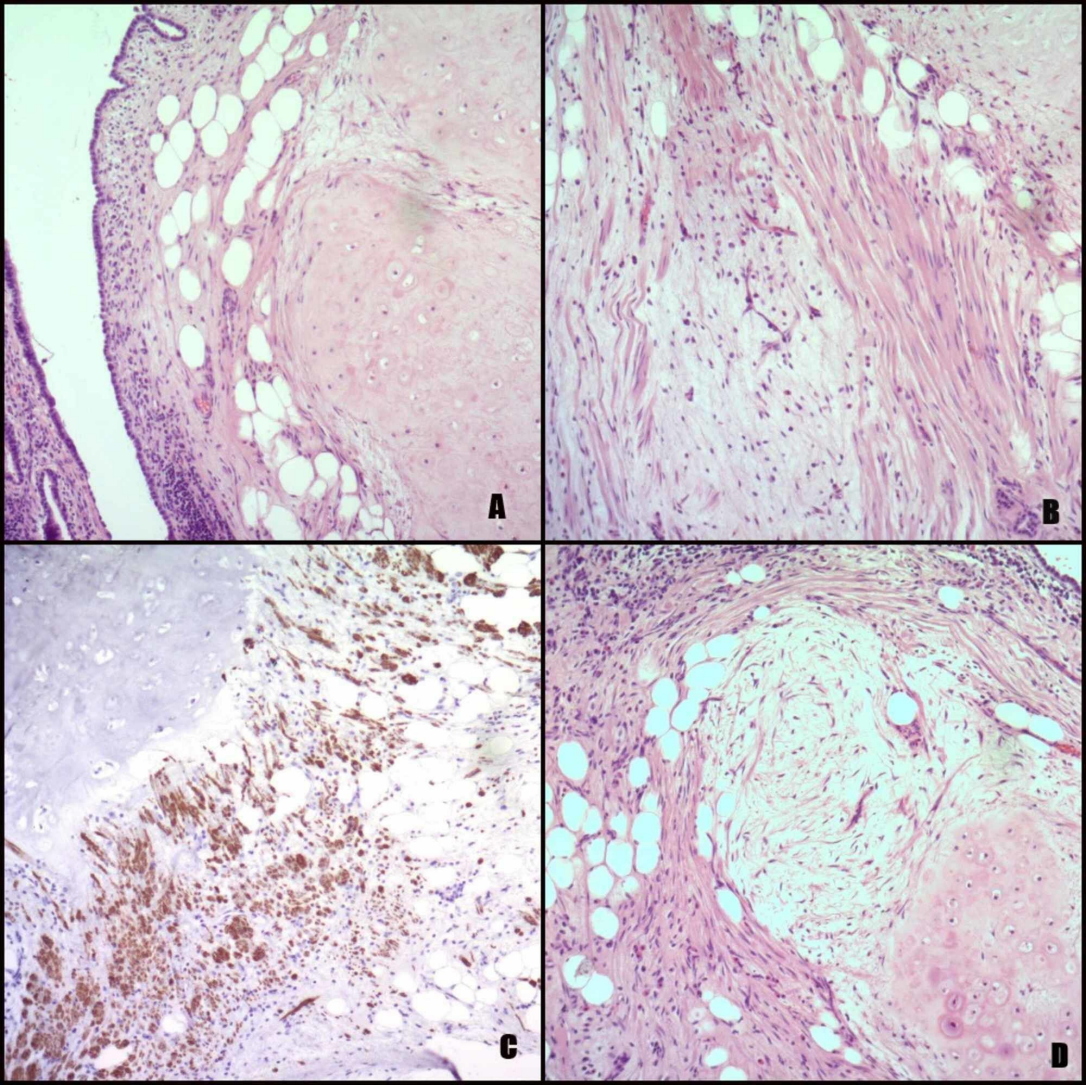
Microphotographs of mediastinal hamartoma (A) Chondroid, adipose, smooth muscle and epithelial tissue (hematoxylin and eosin (HE), x100); (B) Chondroid, fibromyxoid, adipose and smooth muscle tissue (HE, x100); (C) Smooth muscle and adipose tissue (Desmin, x100); (D) Chondroid, adipose, fibromyxoid, smooth muscle and epithelial tissue (HE, x100).

At pathology, the mass was measured to be 4.5 x 3.5 x 3 cm; it had a smooth surface and was rather soft but of elastic consistency. In the cut section, it was yellowish and lobular. Histology showed roughly equal quantities of mature hyaline cartilage and myxoid fibrous and mature adipose tissue. Among those tissues, some smooth muscle fibers were noticed as well as some arborizing slitlike spaces covered with the normal epithelium (Figure 3). The diagnosis of pulmonary hamartoma was made with an unusual medial mediastinal location.

## Discussion

Hamartomas are usually diagnosed as an incidental finding while performing an imaging examination of the thorax for any other reason. They are typically solitary and asymptomatic, although they can compress surrounding lung tissue or produce some other complications (obstruction, infarction, etc.). This important lesion must be considered in differential diagnosis with other solitary lesions, most importantly, the malignancy. On plain X-ray examination, there are not enough characteristic signs to make a correct diagnosis, so the CT-scan is advised. The popcorn-like calcifications are an important clue for diagnosis [2]. They are treated, if large, by surgical resection, and the prognosis is excellent.

Besides the potential harmful effect on the lung tissue, another importance of hamartomas is their association with some diseases, which could have a serious impact on the patients’ health. In one broad study of 24 cases, an unexpectedly high number of patients had some congenital abnormality in other organs; all of them had at least one benign tumor, and in five, a malignant tumor was diagnosed [3]. The occurrence of multiple hamartoma syndrome with Cowden syndrome is well known, but the pulmonary hamartoma is not reported as one of them [3]. In another study, out of 215 patients, 63 (29.3%) had some malignant tumor, and usually, it was lung carcinoma. The direct influence of the occurrence of both lesions is not detected or proven [4].

The most common localization of hamartoma is lung tissue. The mediastinal hamartoma is quite rare, in either the posterior or the anterior part [5-8].

This case is unusual because the previously performed lung imaging examination (in order to diagnose pollen allergy and following exacerbations during many years) did not diagnose any sign of this lesion. Also, the unusual presentation in the medial mediastinum makes the case atypical. Theories about a possible migration of intraparenchymal hamartoma through visceral pleura toward mediastinum are posed [8]. If we accept the theory of neoplastic growth, then we can easily explain both findings in our case: the previously not noticed nodule (in any parenchymal pulmonary part) and the medial mediastinal localization (as the site of primary growth).

## Conclusions

Although a pulmonary hamartoma is not a difficult diagnostic problem at pathology, it must be included in the clinical and radiological differential diagnosis of solitary nodules in the lung parenchyma, and even within the mediastinum.
